# Road terrain recognition based on tire noise for autonomous vehicle

**DOI:** 10.1038/s41598-024-81666-7

**Published:** 2024-12-28

**Authors:** Dongsheng Yang, Dongmin Zhang, Yi Yuan, Zhaoyu Lei, Binlei Ding, Lei Bo

**Affiliations:** New Technology Research Institute, BYD Auto Industry Co., Ltd., Shenzhen, 518118 China

**Keywords:** Road recognition, Tire noise, Deep learning, Mel spectrogram, Classification, Mechanical engineering, Engineering, Mathematics and computing

## Abstract

Effective road terrain recognition is crucial for enhancing the driving safety, passability, and comfort of autonomous vehicles. This study addresses the challenges of accurately identifying diverse road surfaces using deep learning in complex environments. We introduce a novel end-to-end Tire Noise Recognition Residual Network (TNResNet) integrated with a time-frequency attention module, designed to capture and leverage time-frequency information from tire noise signals for road terrain classification. Our method was evaluated on five distinct road types: asphalt, cement, grass, mud, and sand. The performance of TNResNet was rigorously compared against traditional machine learning techniques, including Decision Trees, K-Nearest Neighbors, and Support Vector Machines, as well as advanced deep learning models like Long Short-Term Memory and Convolutional Neural Networks. Experimental results demonstrate that TNResNet achieves superior classification accuracy of 99.48%, outperforming all comparative methods. This work not only establishes a robust framework for road terrain identification but also showcases the significant practical implications of TNResNet in the realm of autonomous vehicle navigation.

## Introduction

The rapid advancements in automated and semi-automated driving technologies have catalyzed the development of advanced driver assistance systems (ADAS), significantly enhancing driver convenience and safety. At the core of these innovations lies vehicle control technology, which is essential for realizing fully automated driving systems ^1–3^. A critical aspect of vehicle control is understanding how different road terrains affect vehicle handling, ride quality, and overall stability ^4^. Accurate information regarding road terrain is crucial for optimizing various vehicle control systems, including electronic stability control (ESC), automatic emergency braking (AEB), and electronic stability program (ESP). Variations in road friction coefficients directly influence tire-road interaction forces, impacting the vehicle’s ability to drive, brake, and steer effectively. Consequently, precise terrain information is necessary for enhancing vehicle performance and safety across diverse environments ^5–7^. Furthermore, a robust road terrain identification system can proactively adjust vehicle settings-such as throttle, transmission, and stability control-during transitions between different surfaces, thereby maintaining control and safety ^8^.

Currently, road terrain recognition techniques primarily include machine vision, vehicle dynamic responses, and tire-pavement interaction noise analysis. Among these, machine vision is the most widely employed method. Notable contributions in this area include a wavelet and support vector machine (SVM)-based approach by Yang et al. ^9^, a convolutional neural network (CNN)-based classification method by Nolte et al. ^10^, and the RTQ-CNN developed by Tumen et al. ^11^. While these machine vision techniques have demonstrated effectiveness, they often face challenges due to environmental disturbances, such as variable lighting and physical obstacles.

In addition, the dynamic responses of vehicles, which are influenced by road roughness and surface materials, provide valuable insights into road conditions. Guan et al. ^12^ proposed a method that focuses on tire cornering stiffness, while Lee et al. ^13^ employed a deep neural network utilizing accelerometer data for real-time classification. Moreover, recent research emphasizes the integration of deep learning approaches for vision sensor-based control in autonomous vehicles ^14^. Despite their promise, these methods frequently encounter challenges in signal acquisition and processing.

Given the limitations of existing methods, there is increasing interest in utilizing tire noise for road terrain recognition. Variations in tire noise intensity across different surfaces present a unique opportunity for analysis. For example, research has shown that tire noise amplitude increases significantly on wet roads ^15^. Techniques such as power spectral density (PSD) ^16,17^ and Mel-frequency cepstral coefficients (MFCC) ^18^ have been employed for feature extraction; however, complex background noise can impede recognition tasks. Recent advancements that incorporate attention mechanisms into network architectures have demonstrated potential in overcoming these challenges ^19^. The works of Xu ^20^ and Zhao et al. ^21^ illustrate the effectiveness of residual networks in tackling complex classification problems.

Additionally, an investigation by Zhong et al. ^22^ explored the relationship between surface texture and friction, emphasizing the importance of surface characteristics in assessing road conditions. This aligns with ongoing efforts to improve skid resistance evaluation through innovative image-based methods ^23^. Furthermore, Khanum et al. ^24^ developed a deep-learning-based network for lane following in autonomous vehicles, showcasing the potential of advanced neural networks to enhance vehicle control systems.

In this study, we introduce the Deep Learning-based Tire Noise Recognition Residual Network (TNResNet), designed to improve the accuracy of road terrain recognition for autonomous vehicles. We conduct a series of experiments to evaluate TNResNet’s performance across various road types, including asphalt, cement, grass, mud, and sand, at different speeds. Our comparisons include established methods such as Decision Trees, K-Nearest Neighbors (KNN), SVM, Long Short-Term Memory (LSTM), and conventional CNNs, ensuring a comprehensive assessment.

The results reveal that TNResNet significantly outperforms comparative methods, achieving an average classification accuracy of 99.48%, which is markedly higher than the best performances recorded for SVM, KNN, and Decision Trees at 86.8%, 78.7%, and 82.7%, respectively. Additionally, TNResNet excels in recall and F1 scores, demonstrating its capacity to minimize false negatives and positives. These findings affirm TNResNet’s effectiveness in recognizing diverse road terrains under complex conditions, leveraging the advantages of deep residual networks and a time-frequency attention mechanism to enhance feature representation and overall model reliability.

## Methodology

In this paper, we propose a time-frequency domain CNN coupled with Mel spectrograms to solve road terrain classification task. The architecture of the TNResNet model, the time-frequency attention mechanism, as well as the process of Mel spectrogram computation will be described in this section.

**Fig. 1:**
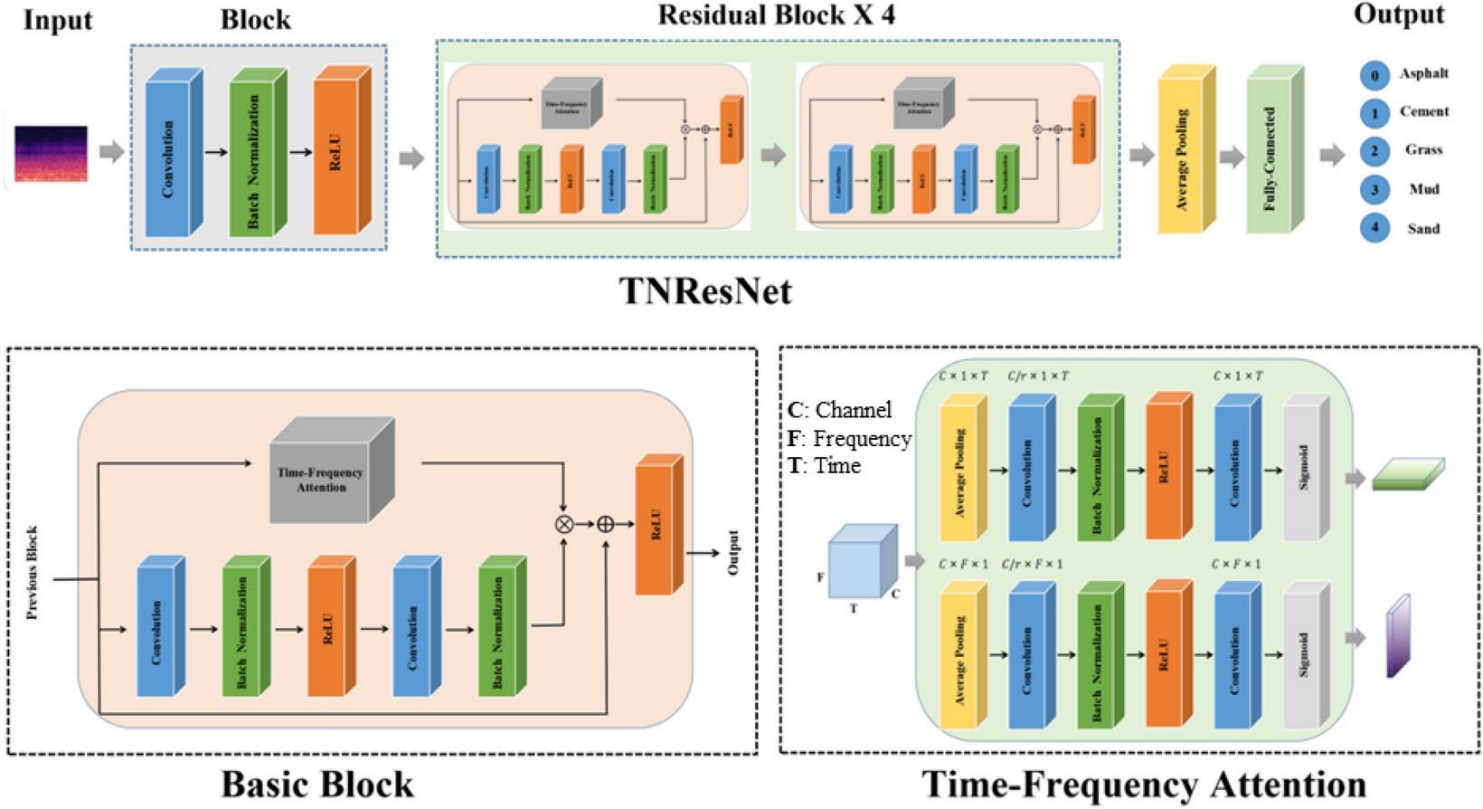
The architecture of the TNResNet.

### Model architecture

The TNResNet model is an innovative adaptation of the ResNet-18 ^25^ architecture tailored for road terrain classification tasks. As shown in Fig. 1, the architecture consists of five blocks. The initial block includes a convolutional layer with a kernel size of 7x7 and a stride of 2, followed by a batch normalization layer and a ReLU activation function. This block is crucial as it extracts initial features from the input Mel spectrograms, enhancing the model’s ability to discern intricate patterns in the data.

The remaining four blocks are structured as residual blocks, each containing two basic blocks. Each basic block consists of convolutional layers with a 3x3 filter size and a stride of 1. The residual connections in these blocks allow for more effective training by mitigating the vanishing gradient problem, thus improving model convergence. The design rules followed are: (i) When the output feature map’s size matches the input, the number of filters equals the number of input feature maps. (ii) When the output feature map size is halved, which is achieved using convolutional layers with a stride of 2, the number of filters is doubled.

Each convolutional layer is succeeded by batch normalization and ReLU activation layers. Additionally, we implement a time-frequency attention layer to effectively extract and emphasize time and frequency information from the input feature map, contributing to the model’s adaptability to varying conditions. The architecture concludes with a global average pooling layer and a fully-connected layer equipped with a softmax activation function for classification.

The feature map is obtained by a convolution operation as Eq. 1 between input data and convolutional kernel.

1 $$\begin{aligned} y_{k} = \sum _{u = 1}^{N_{h}}{\sum _{v = 1}^{N_{w}}{\sum _{w = 1}^{N_{c}}x}} \bullet \ \omega _{u,v,w,k} + b_{k} \end{aligned}$$

where $y_{k}$ denotes $k^{\textrm{th}}$ feature map; $N_{h}$ and $N_{w}$ are the convolutional kernel height and width, respectively; $N_{c}$ denotes the number of input feature map; $b_{k}$ is the bias for $k^{\textrm{th}}$ convolutional kernel; and $\omega$ is the learnable weight of the convolutional kernel. The output of convolutional layer is fed to the batch normalization which is applied to reduce internal co-variate shift for accelerating deep network training. The computation process of batch normalization can be summarized as:

2 $$\begin{aligned} \mu _{\textrm{B}}&=\frac{1}{m} \sum _{i=1}^m x_i \nonumber \\ \sigma _B^2&=\frac{1}{m} \sum _{i=1}^m\left( x_i-\mu _B\right) ^2 \nonumber \\ x_i&=\frac{x_i-\mu _B}{\sqrt{\sigma _B^2+\varepsilon }} \nonumber \\ y_i&=\gamma \cdot x_i+\beta \end{aligned}$$

where *x* denotes input feature map, $\gamma$ and $\beta$are learnable parameters, $\varepsilon$ is a constant value to prevent the denominator from being zero. In general, the batch normalization layer is followed by an activation function layer. The activation functions are usually defined as nonlinear functions to deal with complex nonlinear problem and improve the expressiveness of the model. The most widely used activation function is ReLU. After above operations, the final feature map (3-dimensional) is flattened to an 1-dimensional feature vector to fed the fully connected layer which has as many nodes as the number of classes for classification. In order to obtain probability of each class the deep neural network generally have a softmax output layer which is used the softmax function defined as Eq. 3. After get the probability of each class, the model selects the output class that has the greatest possibility, as shown in Eq. 4

3 $$\begin{aligned} P_{i}= & \frac{\exp \left( o_{i} \right) }{\sum _{k = 1}^{n}{\exp \left( o_{k} \right) }} \end{aligned}$$

4 $$\begin{aligned} y_{out}= & \max {P_1,P_2,\cdot ,P_{n-1},P_n} \end{aligned}$$

where $o_i$ denotes the output value of $o^{th}$ node in the fully connected layer, and $P_i$ denotes the probability value for $i^{th}$ class.

### Time-frequency attention

**Fig. 2:**
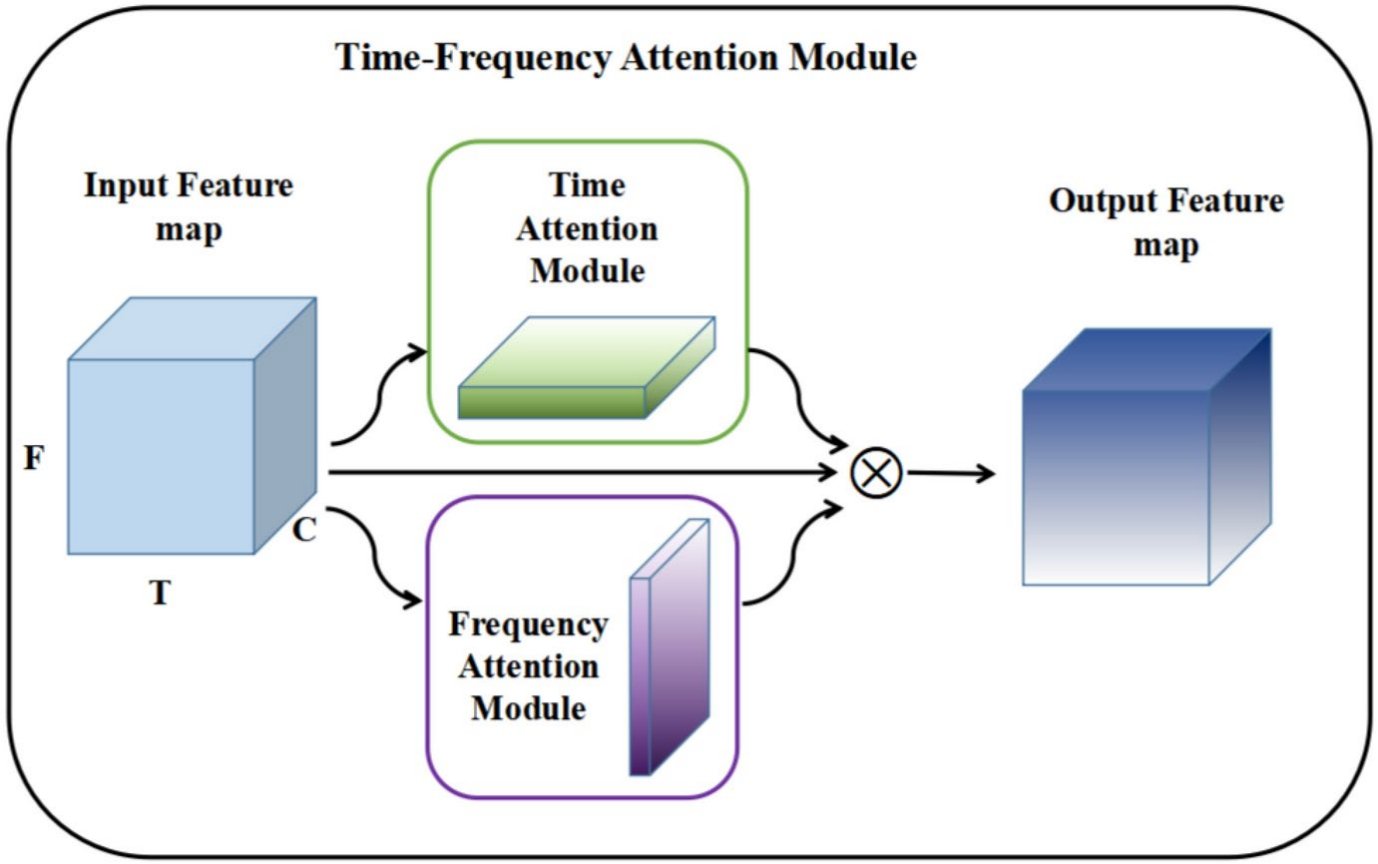
The overview of time-frequency attention module.

We propose a time-frequency attention module inspired by the convolutional block attention module (CBAM) [34] module. For a given input feature map $F \in \mathbb {R}^{C \times F \times T}$, the time-frequency attention module sequentially infers a time-weighted feature map $F_{T} \in \mathbb {R}^{C \times 1 \times T}$ and a frequency-weighted feature map $F_{F} \in \mathbb {R}^{C \times F \times 1}$ as illustrated in Fig. 2.

### Model architecture

The overall attention process can be represented as:

5 $$\begin{aligned} F_T&=M_T(F) \nonumber \\ F_F&=M_F(F) \nonumber \\ F_{\text {out }}&=F \otimes F_T \otimes F_F \end{aligned}$$

in which $F_{T}$, $F_{F}$ and $F_{out}$ are the output of time attention module, frequency attention module, and final output of time-frequency attention module, respectively. And the symbol “$\otimes$” denotes element-wise multiplication, in which the weight values are broadcasted during multiplication: time-weighted values are broadcasted along frequency axis, while frequency-weighted values are broadcasted along time axis.

**Fig. 3:**
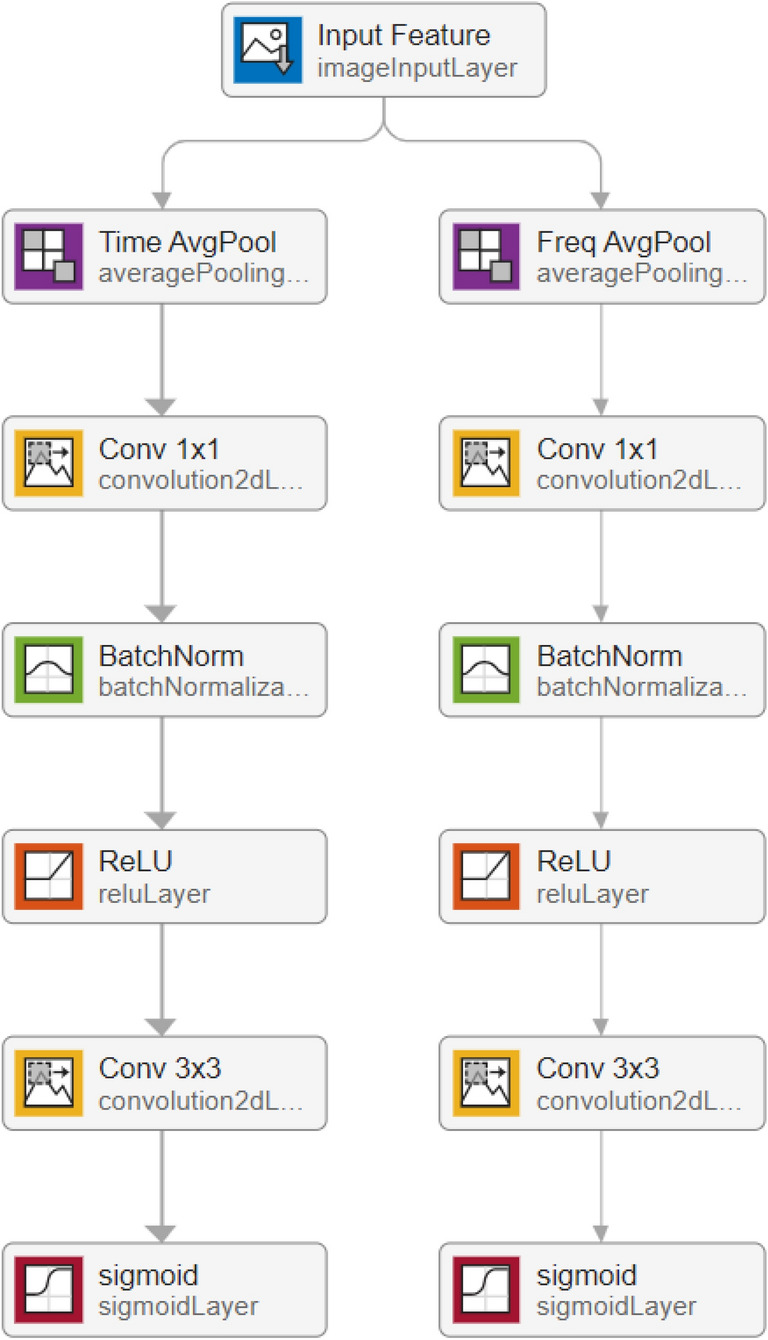
Time-frequency attention module for computing time-weighted and frequency-weighted feature map.

The computation process of time-weighted and frequency-weighted feature map are depicted in Fig. 3. The time-weighted feature map focuses on the meaningful moments for a given input feature map, and can be generated by utilizing the inter-time relationship of feature map. We first apply average-pooling operation along the frequency axis. To reduce parameter overhead, we apply a 1x1 kernel size convolutional layer for reducing dimensions to produce a feature map $F_{m} \in \mathbb {R}^{C/r \times 1 \times T}$, and batch normalization and ReLU activation function followed by the 1x1 kernel size convolutional layer. On the concatenated feature descriptor, we apply a 3x3 kernel size convolutional layer and Sigmoid activation function to generate a time-weighted feature map $F_{T} \in \mathbb {R}^{C \times 1 \times T}$ that encodes which moments to emphasize or suppress. In summary, the time-weighted map is computed as:

6 $$\begin{aligned} F_T=f_{\text {sigmoid }}\left( f_{\text {conv }}^{3 \times 3}\left( f_{\operatorname {ReLU}}\left( f_{\textrm{BN}}\left( f_{\text {conv }}^{1 \times 1}\left( f_{\text {avgpool }}^F(F)\right) \right) \right) \right) \right) \end{aligned}$$

where *F* denotes input feature map, $f^{F}_{\textrm{avgpool}}$ denotes average-pooling operation along the frequency axis, $f_{BN}$ denotes batch normalization operation, $f_{\text {conv}}^{1 \times 1}$ and $f_{\text {conv}}^{3 \times 3}$ represent convolution operations with filter size of $1\times 1$ and $3\times 3$, $f_{\textrm{ReLU}}$ and $f_{\textrm{sigmoid}}$ represent ReLU activation function and Sigmoid activation function, respectively. The frequency-weighted feature map is mostly similar to the time-weighted feature map but focuses on meaningful frequency for a given input feature map, which is computed as

7 $$\begin{aligned} F_F=f_{\text {sigmoid }}\left( f_{\text {conv }}^{3 \times 3}\left( f_{\operatorname {ReLU}}\left( f_{\textrm{BN}}\left( f_{\text {conv }}^{1 \times 1}\left( f_{\text {avgpool }}^F(F)\right) \right) \right) \right) \right) \end{aligned}$$

in which $f^{T}_{\textrm{avgpool}}$ denotes average-pooling operation along the time axis.

### Mel spectrogram

Spectrogram is a graphical representation that visualizes the time-frequency characteristics of a signal. It shows the frequency content of a signal over time and is commonly used to analyze how a signal changes in time and frequency. There are several ways to obtain a spectrogram in digital signal processing, such as generating by an optical spectrometer, a bank of band-pass filters, Fourier transform, and a wavelet transform, etc. In this work, we compute spectrogram *S* by the short-time Fourier transform (STFT) ^26^ of a tire noise audio signal. Since the frequency of the tire noise signal is mainly concentrated in the range of 20 - 5000 Hz, in order to show the low-frequency information more clearly, we log-convert its energy value on the basis of the spectrogram:

8 $$\begin{aligned} S=\log _{10}\left( |X(\tau , \omega )|^2+\varepsilon \right) \end{aligned}$$

where $\varepsilon$ is added to prevent the log value infinite, and $X_{\tau ,\omega }$denotes magnitude and phase of basis sinusoidal frequencies $\omega$ at different time points $\varepsilon$, which is obtained by STFT as

9 $$\begin{aligned} X(\tau , \omega )=\sum _{n=-\infty }^{+\infty } x[n] \cdot w[n-\tau ] \textrm{e}^{-\textrm{j} \omega n} \end{aligned}$$

in which *x* denotes input time-domain signal, and *w* denotes window function which can reduce spectral leakage caused by the sub-framing. Here the Hamming window as Eq. 9 is used to provide a narrower main flap in the frequency domain, which helps to concentrate the energy of the signal,

10 $$\begin{aligned} w(k)=a_0-a_1 \cos \left( \frac{2 k \pi }{N-1}\right) \end{aligned}$$

where $$a_{0} = 0.54$$ and $$a_{1} = 0.46$$.

Mel-frequency analysis begins by converting linear frequency to Mel frequency which is given by Eq. 10, and averaging the bandwidth occupied by the signal within the Mel frequency to obtain a set of filters. Each filter in the filter bank is triangular, with a response of 1 at the center frequency and decreasing linearly to 0 until it reaches the center frequency of two adjacent filters:

11 $$\begin{aligned} m \cdot =2595 \cdot \log _{10}\left( 1+\frac{f}{700}\right) \end{aligned}$$

where *m* and *f* denote Mel frequency and linear frequency, respectively. For a balance of time and frequency resolution, we split the input tire noise signal into frames of 50 ms length. The corresponding overlap between subsequent frames is 40 ms length. In our work, the input tire noise signal of *t* seconds is converted into a sequence of 64-dimensional log Mel-filter bank features computed with a 50 ms Hamming window every 10 ms. The results are a series of 96*t* $\times$ 64 Mel spectrograms as input to the TNResNet.

## Experiment

### Datasets

**Fig. 4:**
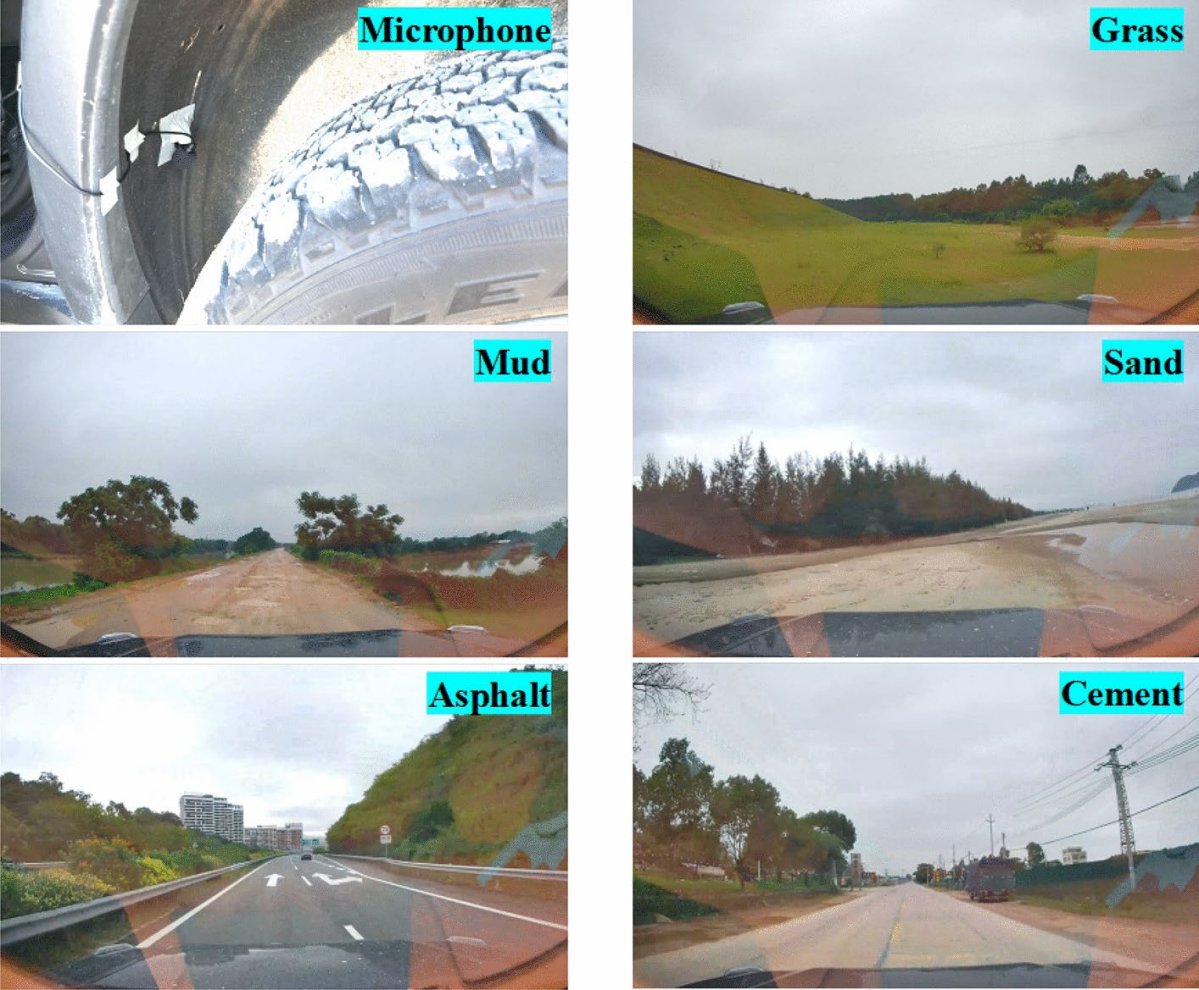
Type of road to be inspected for data collection.

As suggested by Lee et al. ^13^, the testing vehicle was instrumented with a single microphone installed on the front wing of the right-rear wheel proximity, which permits to achieve a better signal to noise ratio (SNR), and prevents noise disturbance from the vehicle’s engine, exhaust pipe and other parts. In addition, to avoid the influence of wind, a windscreen was used for the microphone. We used a microphone with 44.1 kHz sampling frequency to collect tire noise signal driving on asphalt road, cement road, mud road, grass road, and sand road. In addition, the driving speed is controlled from 10 to 80 km/h with a 10 km/h interval,as shown in Fig. 4. The time length for different roads under varying speed segments are shown in the Supplementary Table S1.

### Model training

**Data augmentation**: Data augmentation is a useful technique to increase variability in the training data and prevent over-fitting. In this work, we apply time pitch shift, time inversion ^27^, time mask and mix up ^28^ to augment data during training:

1. Pitch shift. This method allows to adjust pitch of audio depended on the frequency. Therefore, it can be viewed as a scale shift of the frequency;
2. Time inversion. Time inversion is an effective data augmentation technique that is related to random horizontally flip of images during the training on the visual classification datasets;
3. Time mask. Time mask is applied such that t consecutive time interval $\left[ t_0, t_0 \cdot +\cdot t\right]$ are masked, where t - is chosen from a uniform distribution from 0 to a time mask parameter *t*, and t 0 is chosen from $[0, T-t]$, where *T* is the number of sample points for the audio. There can be more than one time mask in each audio. The time mask can improve the robustness of the TNResNet to time distortion of audio clips.
4. Mix up. Mix up is a way to augment datasets by interpolating two audios of identical target. For instance, we denote the input of two audios as $x_1$ and $x_2$ which have the same target. Then, the augmented input can be obtained by $x=a x_1+(1-a) x_2$, where *a* is hyper-parameter.

**Optimizer**: We follow and adapt Ruder’s summary ^29^of the state-of-the-art optimization algorithms. For training a deep or complex neural network, adaptive moment estimation (Adam) is used to compute adaptive learning rates for each parameter. Adam is designed to combine the advantages of Adadelta and RMSprop, and keep an average exponential decay rate of past gradients like momentum. The formula for updating Adam’s weights is as follows:

12 $$\begin{aligned} g_t&=\nabla _\theta f_t\left( \theta _{t-1}\right) \nonumber \\ m_t&=\beta _1 \cdot m_{t-1}+\left( 1-\beta _1\right) \cdot g_t \nonumber \\ v_t&=\beta _2 \cdot v_{t-1}+\left( 1-\beta _2\right) \cdot g_t^2 \nonumber \\ \widehat{m}_t&=\frac{m_t}{\left( 1-\beta _1^t\right) } \nonumber \\ \hat{v}_t&=\frac{v_t}{\left( 1-\beta _2^t\right) } \nonumber \\ \theta _t&=\theta _{t-1}-\alpha \frac{\widehat{m}_t}{\sqrt{\hat{v}_t}+\epsilon } \end{aligned}$$

where *a* denotes learning rate, $g_{t}$ denotes the gradient of the current parameter, $\beta _1$ and $\beta _2$denote the exponential decay rates of the first and the second moment estimate, respectively. In this work, $\beta _{1}$and $\beta _{2}$ are defined as 0.9 and 0.999.

**Loss function**: For a classification task, cross entropy is usually employed as its loss function which can be expressed by the following equation,

13 $$\begin{aligned} Loss(j) = \ - \sum _{i = 1}^{K}{y_{i,j}\log \left( p_{i} \right) } \end{aligned}$$

where *K* denotes the number of road terrain classes, $y_{i,j}$ equals 1 if $i = j$ and otherwise 0, $p_{i}$ denotes the possibility when the prediction is for label *i*.

**Fig. 5:**
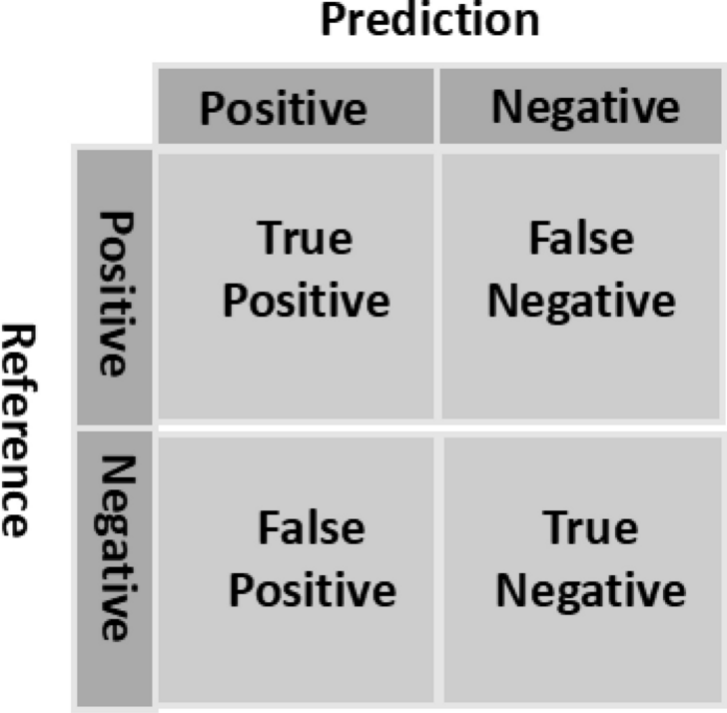
Confusion matrix.

**Metrics**:

To rigorously assess the effectiveness of a multi-class classifier, it is essential to employ a comprehensive suite of metrics that can holistically capture the model’s performance. The chosen metrics—accuracy, precision, recall, and F1-score—are derived from the confusion matrix, as illustrated in Fig. 5. These metrics are critically selected because they provide a balanced evaluation of the classifier’s performance across various aspects:

Accuracy measures the overall ability of the model to correctly predict both positive and negative classes. In the context of autonomous vehicle terrain recognition, a high accuracy indicates that the model can reliably differentiate between various types of road surfaces. This capability is crucial for minimizing the risk of erroneous navigational and control decisions in practical applications. By ensuring high accuracy, the model aids in enhancing the operational reliability of autonomous driving systems, contributing to safer and more efficient vehicle operations.

14 $$\begin{aligned} \text { accuracy } = \frac{T P+T N}{T P+F P+T N+F N} \end{aligned}$$

Precision quantifies the accuracy of the model when it predicts positive outcomes. In the realm of autonomous driving vehicle terrain recognition, high precision means that when the model predicts a certain type of road surface, it is likely correct. This is crucial for avoiding unnecessary or incorrect vehicle responses to perceived road conditions, which could compromise the vehicle’s safety and operational efficiency. High precision ensures that the autonomous system’s adjustments to vehicle dynamics-like speed adaptation and stability control-are based on accurate detections, minimizing the likelihood of inappropriate reactions that could lead to hazardous driving situations.

15 $$\begin{aligned} \text {precision } = \frac{T P}{T P+F P} \end{aligned}$$

where *TP*, *FP*, *FN* and *TN* are true positive, false positive, false negative and true negative, respectively.

Recall, also known as the true positive rate, measures the proportion of actual positives that the model correctly identifies. For autonomous driving systems, a high recall is essential as it signifies the model’s ability to recognize most of the actual road terrains. This capability is particularly critical to prevent safety issues that could arise from unrecognized road conditions. Ensuring high recall helps in reducing the chances of accidents caused by inadequate response to actual road terrains, thereby enhancing the predictive safety features of the vehicle.

16 $$\begin{aligned} \text { recall } = \frac{T P}{T P+F N} \end{aligned}$$

F1-Score is the harmonic mean of precision and recall, providing a balance between these two metrics. It is particularly useful in scenarios where there is an imbalance among classes, as it takes into account both the precision and the coverage of the positive class by the model. The F1 score is a critical metric in autonomous driving applications where missing a road type (low recall) or falsely identifying a road type (low precision) could have severe implications. By optimizing for a high F1 score, developers can achieve a balanced model that performs well under varied and unpredictable environmental conditions, which is paramount for the adaptive response systems of autonomous vehicles.

17 $$\begin{aligned} F1_{Score} = 2\frac{{\text {precision}\cdot \text {recall}}}{\text {precision} + \text {recall} } \end{aligned}$$

We implement TNResNet in MATLAB, and the model was trained with the batch size of 128 for 1000 iterations. Adam is used as the optimizer with initial learning rate of 0.001 and decreased by a factor 0.1 for every 400 iterations. We evaluate the results using accuracy, precision, recall and F1-score. All the experiments are performed using NVIDIA GeForce RTX 3080 with 10 GB memory.

**Fig. 6:**
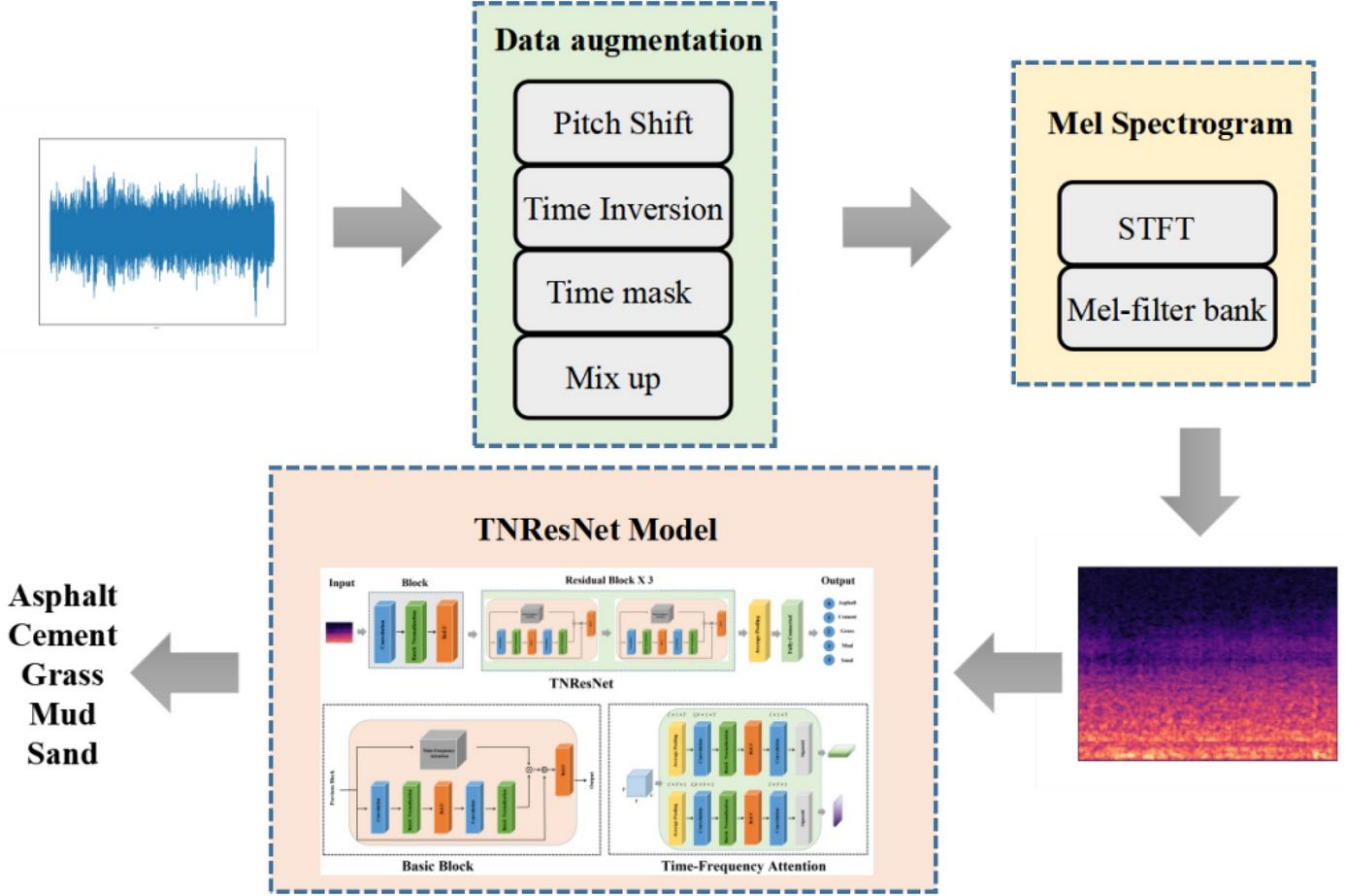
Workflow diagram for road terrain recognition.

The input of TNResNet is the Mel spectrogram, and the output is the prediction label of road terrain type. We have collected tire noise signals of different road terrains and extracted the Mel spectrogram from original signals. To improve the training accuracy and prevent over-fitting, we use pitch shift, time inversion, time mask and mix up to expand the tire noise signals. The workflow diagram for road terrain recognition is shown as Fig. 6.

**Fig. 7:**
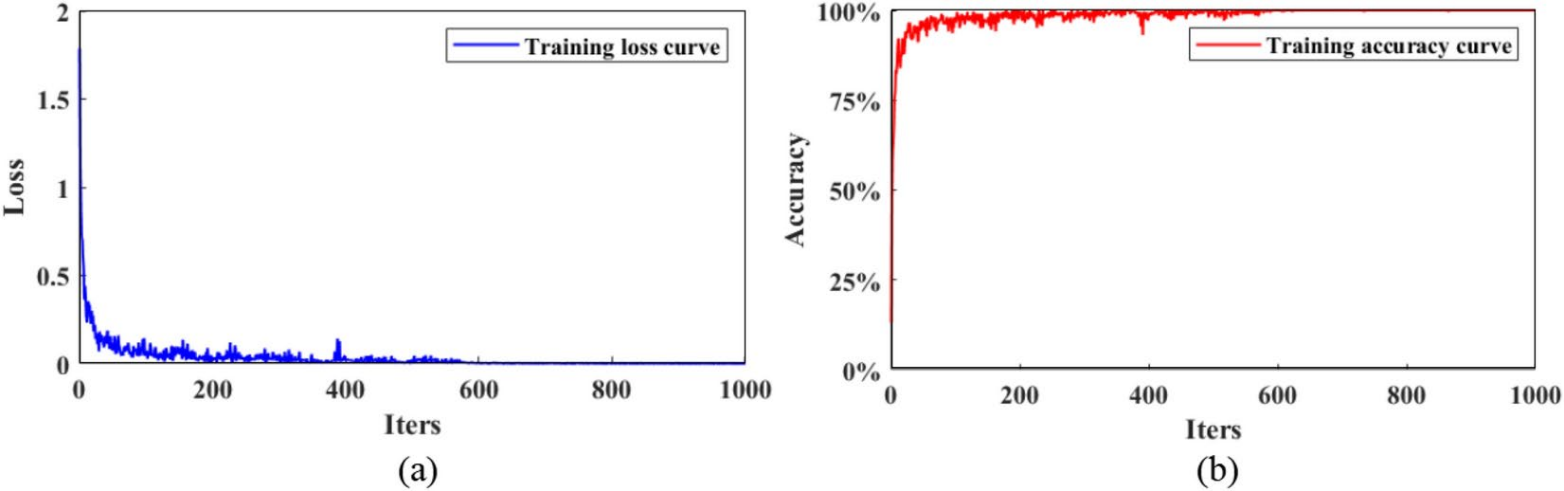
The evolutions of (**a**) training loss and (**b**) training accuracy of TNResNet model with iterations. After 600 iterations the model tends to be stable.

The training samples contain two parts: 80% training data and 20% validation data. As the training time increases, the model tends to be stable after iterations 600, where the training loss of the model decreases to 0, and the training accuracy of the model increases to 100%, shown as Fig.  7.

**Fig. 8:**
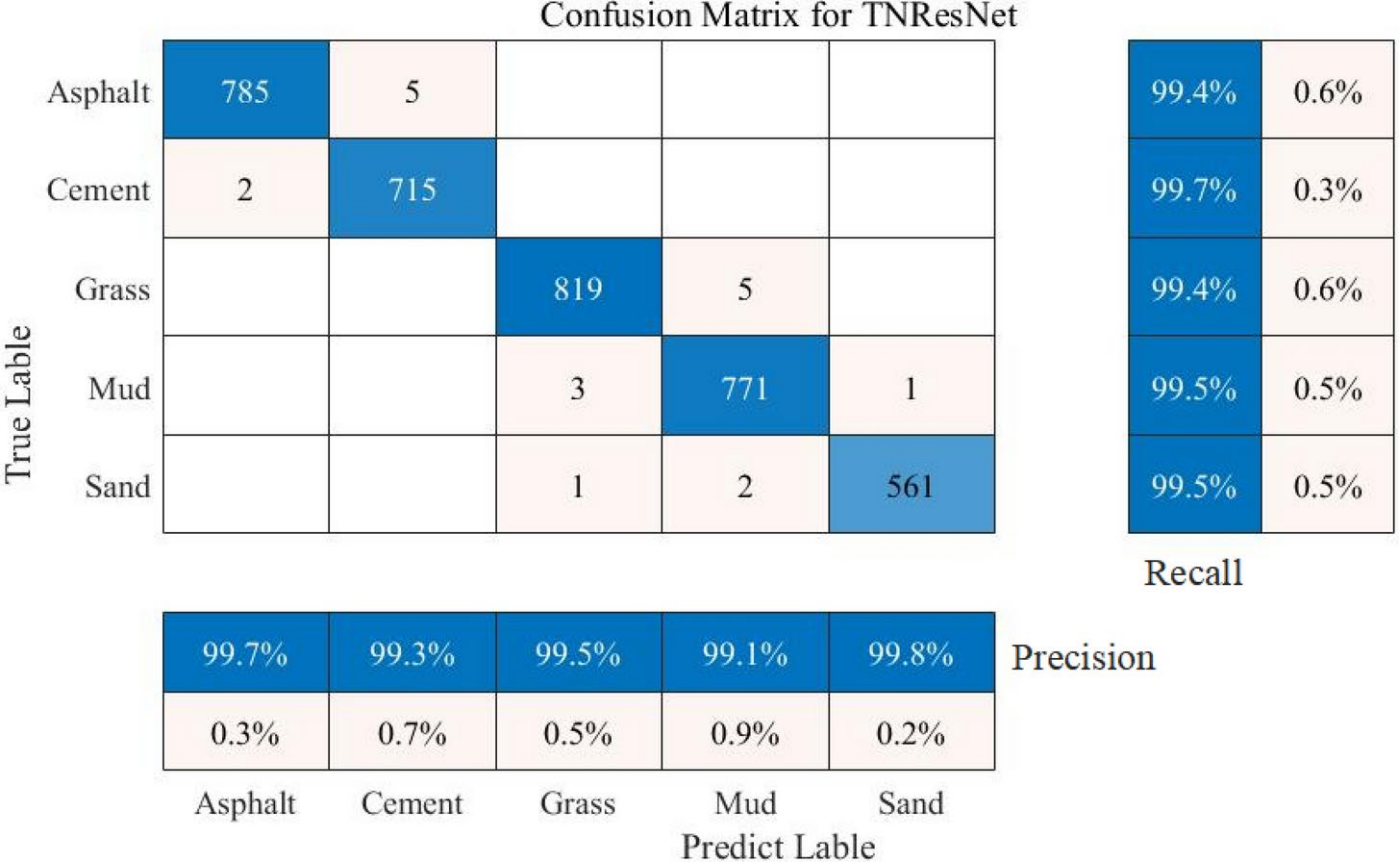
The confusion matrix of TNResNet model validation.

We use 3670 validation samples to verify the prediction accuracy of the model and the classification accuracy of all the validations is 99.48%. The average recognition precision, recall and F1-score are 99.48%, 99.50% and 99.46%, respectively. More details of the validation results can be seen in Fig. 8 and Tab. 1.Each sample contains 5000 sampled signal points and the number of sampled signal points remains consistent across speeds and road conditions. To increase transparency, specific data for some of the experimental samples are provided in the appendix.Fig. 8 illustrates the signal data for different road conditions, with confidence labels derived by analysing the probability distributions of the model outputs. Specifically, the model’s predictive confidence for each road type is determined by its corresponding output probability.

**Fig. 9:**
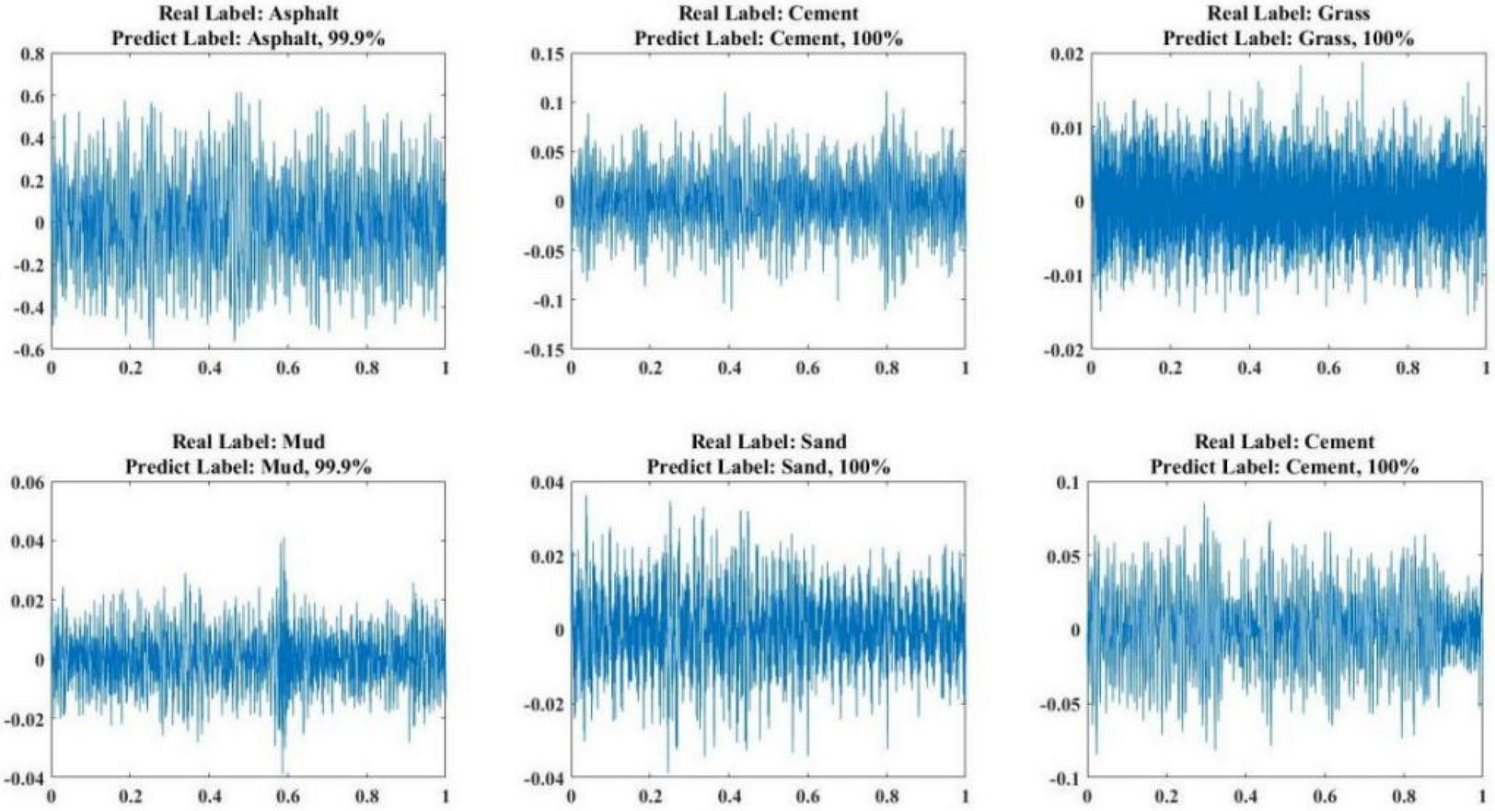
Valuate results of TNResNet contain label of road type and confidence.

Different road terrains are selected to evaluate the trained TNResNet, the valuate results of the model are shown in Fig 9. The prediction result of the model contains label and confidence, and the confidence is the probability of the current prediction label.

**Table 1:** Evaluation metrics results of the TNResNet.

Classes	Precision	Recall	F1-score
Asphalt	99.7%	99.4%	99.5%
Cement	99.3%	99.7%	99.5%
Grass	99.5%	99.4%	99.4%
Mud	99.1%	99.5%	99.3%
Sand	99.8%	99.5%	99.6%

### Comparison with machine learning

Machine learning methods can be used in related studies about tire noise signals. Different with deep learning approaches, machine learning methods require pre-processing step to extract high-level features from the tire noise signals, which will represent the patterns to be recognized. Among these features, we extracted 12 kinds of common signal features from time domain and frequency domain which are attached in Supplementary Table S2, And here we will compare our TNResNet model with 3 machine learning methods, including decision tree (DT), KNN and SVM.

**Fig. 10:**
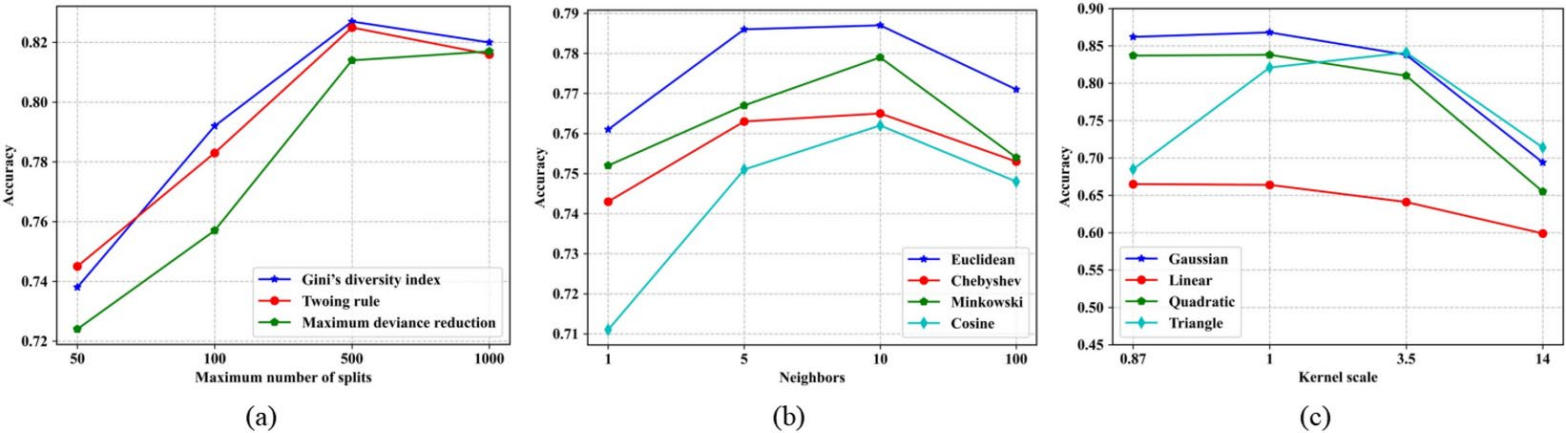
Validation accuracy curves for three machine learning methods: (**a**) DT, (**b**) KNN and (**c**) SVM. The highest validation accuracy for DT, KNN and SVM is 82.7%, 78.7% and 86.8%, respectively.

DT is a supervised learning method applied in classification, which is a kind of tree structure, each internal node represents a judgement on a feature, each branch represents the output of a judgement and finally, and each leaf node represents a classification result. In the method, each sample has a set of features and the prediction is obtained by making judgement on each feature. Here, 3 split criterions were analyzed including Gini’s diversity index, twoing rule and maximum deviance reduction. Shown as Table 2 and Fig. 10(a), when the maximum number of splits does not exceed 500, the more splits, the greater the accuracy values for DT. And when using Gini’s diversity index as split criterion, DT model has the highest validation accuracy reaching 82.7% when the maximum number of splits is 500.

**Table 2:** Validation accuracy values for DT.

Split criterion	Maximum number of splits				Average
	50	100	500	1000	
Gini’s diversity index	73.8%	79.2%	82.7%	82.0%	79.4%
Twoing rule	74.5%	78.3%	82.5%	81.6%	79.2%
Maximum deviance reduction	72.4%	75.7%	81.4%	81.7%	77.8%
Average	73.7%	77.7%	82.2%	81.8%	78.8%

KNN is a classification method based on similarity metrics between samples to recognize patterns. For a new sample, the distance from the sample to each training sample is calculated, identifying the *k* nearest neighbors. The new sample class is obtained by the most common class among the *k* neighbors. Here, KNN models with 1, 5, 10, 100 neighbors and Euclidean, Chebyshev, Minkowski, Cosine distances were analyzed for the optimal neighbor’s value and distance type. For the KNN technique, the best model is using Euclidean distance as similarity metric with 10 neighbors, whose validation accuracy reaches 78.7%, shown in Table 3 and Fig. 10(b).

**Table 3:** Validation accuracy values for KNN

Distance	Neighbors				Average
	1	5	10	100	
Euclidean	76.1%	78.6%	78.7%	77.1%	77.6%
Chebyshev	74.3%	76.3%	76.5%	75.3%	75.6%
Minkowski	75.2%	76.7%	77.9%	75.4%	76.3%
Cosine	71.1%	75.1%	76.2%	74.8%	74.3%
Average	74.2%	76.7%	77.3%	75.7%	76.0%

SVM is also a supervised learning method for classification, where the technique searches for an optimal hyperplane that splits the samples classes. For non-linear problem, it is necessary to use the kernel hyper-parameter, which converts a non-separable problem into a separable problem. Here, models for 4 kernel function were analyzed: Gaussian, linear, quadratic and triangle. For the SVM technique shown in Table 4 and Fig. 10(c), the linear kernel function obtained the worst validation accuracy values. The kernel scale has much influence on the final result, with larger kernel scale, the results tend to get worse. In addition, the best model is using Gaussian kernel function with kernel scale 1, reaching 86.8% validation accuracy.

**Table 4:** Validation accuracy values for SVM.

Kernel function	Kernel scale				Average
	0.87	1	3.5	14	
Gaussian	86.2%	86.8%	83.8%	69.4%	81.6%
Linear	66.5%	66.4%	64.1%	59.9%	64.2%
Quadratic	83.7%	83.8%	81.0%	65.5%	78.5%
Triangle	68.5%	82.1%	84.1%	71.4%	76.5%
Average	76.2%	79.8%	78.3%	66.6%	75.2%

**Fig. 11:**
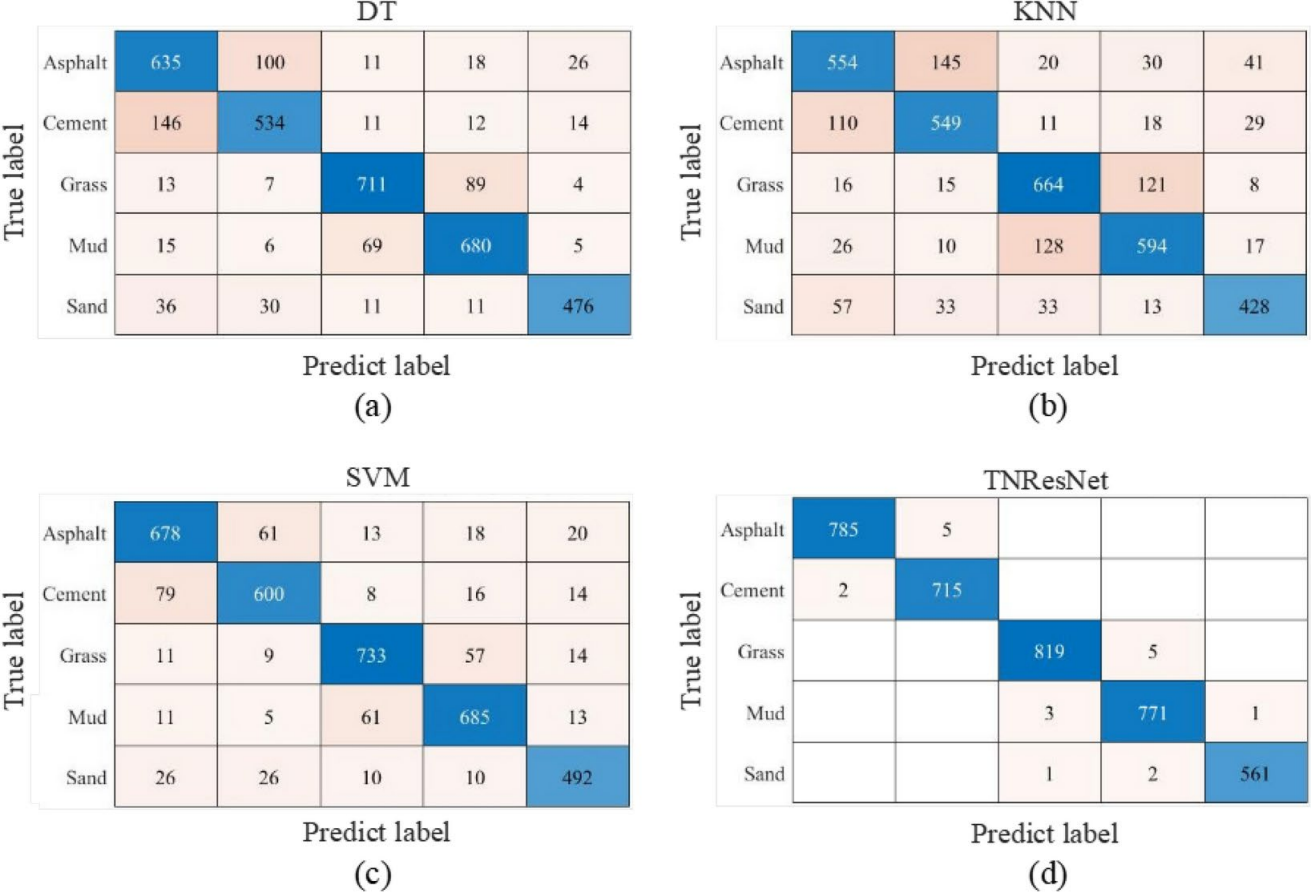
Confusion matrix for each best model of machine learning methods and TNResNet: (**a**) DT; (**b**) KNN; (**c**) SVM; (**d**) TNResNet. The TNResNet method shows robust and accurate prediction.

**Fig. 12:**
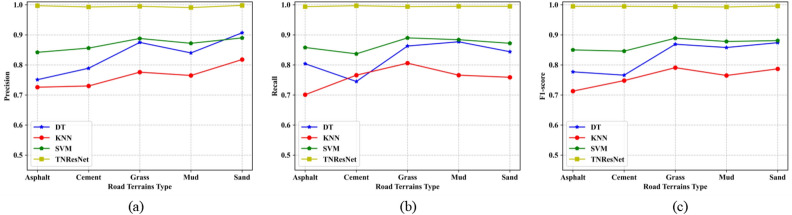
Evaluation metrics curve for each best model of machine learning methods and TNResNet: (**a**) precision; (**b**) recall; (**c**) F1-score. The TNResNet shows a much better performance than DT, KNN and SVM.

To evaluate the performance of the proposed TNResNet model, the confusion matrix for each best model of machine learning methods and TNResNet were calculated and are shown as Fig. 11, which displaies the robust and high accuracy of the proposed TNResNet method. More details could be found in Fig. 12 and Supplementary Table S3, where the accuracy, precision, recall and F1-score were calculated for each terrain on the same datasets, it’s apparent that the TNResNet has a much better performance than DT, KNN and SVM, improving 16.2%, 23.2%, 12.5% in average precision; 16.8%, 23.5%, 12.7% in average recall; and 16.6%, 23.4%, 2.6% in average F1-score, respectively.

### Comparison with deep-learning

Effective feature extraction of signals is a complex problem in the application of machine learning techniques, and sometimes, it is very difficult to extract high-level features. But this problem has been mostly solved by deep learning models composed of multiple processing layers to the data high-level representation. Speech recognition, audio classification and many other applications have been significantly improved based on deep learning models. To make comparison, we built and analyzed long short-term memory (LSTM) and CNN models, which were trained the same as TNResNet, using Adam optimizer and cross entropy as loss function.

**Fig. 13:**
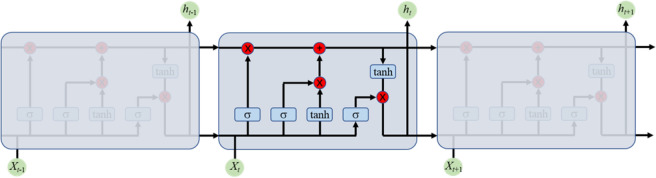
The architecture of LSTM.

LSTM is a specific form of recurrent neural network (RNN). Compared to RNNs, LSTM can better solve the gradient vanishing and gradient explosion problems during long sequence training. In this technique, the combination of three gating units (input gate, forgetting gate and output gate) determines the out of each time step, shown as Fig. 13. This structure enables LSTM to learn long-term dependencies. The input gate controls the current state information, the forgetting gate controls how much of the history information is retained, and the output determines which information will eventually be outputted. We analyzed several LSTM-based models both in unidirectional and bidirectional forms as listed in Table 5, in which LSTM 6 composed of an input layer, three recurrent and regularization blocks and a block of fully connected layers for output production is the best model with 96.40% validation accuracy, and bidirectional LSTM has superior performance than unidirectional LSTM.

**Table 5:** Validation accuracy values for LSTM-based models.

Models	Accuracy	Layers
LSMT 1	94.34%	1 LSTM with 256 units, 1 Dropout of 0.2, 1 Fully-connect layer of 256 units and ReLU activation
LSTM 2	94.89%	Same as LSTM 1, but LSTM are bidirectional
LSTM 3	95.29%	3 blocks of LSTM with 256 units and Dropout of 0.2, 1 Fully-connect layer of 256 units and ReLU activation
LSTM 4	95.87%	Same as LSTM 3, but LSTM are bidirectional
LSTM 5	96.12%	3 blocks of LSTM with 256 units, Batch Normalization and Dropout of 0.2, 1 Fully-connect layer of 256 units and ReLU activation
LSTM 6	96.40%	Same as LSTM 5, but LSTM are bidirectional

CNN can extract features directly from tire noise signal, which is inspired by the mechanism of the biological receptive field and is specifically designed to process data with a grid-like structure, such as image. The tire noise signal was transformed into a RGB image by using CWT as the input of CNN-based models. The convolutional layer is the most important layer for CNN, each convolutional layer has *n* filters with a kernel size of *m*. Convolution layer is usually followed by pooling and fully connected layers, which are used to perform dimension reduction and integrate the extracted features. For the CNN-based approach, we analyzed several network models using different pooling layers as listed in Table 6, from which it can be found that the average pooling is better than max pooling and CNN 3 is the best model with 95.20% validation accuracy. CNN 3 is composed of three blocks of convolution, batch normalization, ReLU activation and average pooling, and a block of fully connected layer. The feature is extracted by convolutional layers with kernel size of 3, which has 16, 32 and 64 filters respectively. Batch normalization is used to standardize the inputs for regularization, and average pooling 2D layer is used to down-sampling for accelerating training and avoiding over-fitting. Finally, a block of fully connected layers for output production.

**Table 6:** Validation accuracy values for CNN-based models.

Models	Accuracy	Layers
AIM ^25^	93.11%	2 blocks of Conv2D with 16-32 filters, kernel 3, batch normalization, ReLU activation and average pooling 2D
CNN 2	93.08%	Same as AIM, but the pooling operations of blocks are max pooling 2D
CNN 3	95.20%	3 blocks of Conv2D with 16-32-64 filters, kernel 3, batch normalization, ReLU activation and average pooling 2D
CNN 4	94.70%	Same as CNN 3, but the pooling operations of blocks are max pooling 2D
CNN 5	95.10%	3 blocks of Conv2D with 32-64-128 filters, kernel 3, batch normalization, ReLU activation and average pooling 2D
CNN 6	94.82%	Same as CNN 5, but the pooling operations of blocks are max pooling 2D

**Fig. 14:**
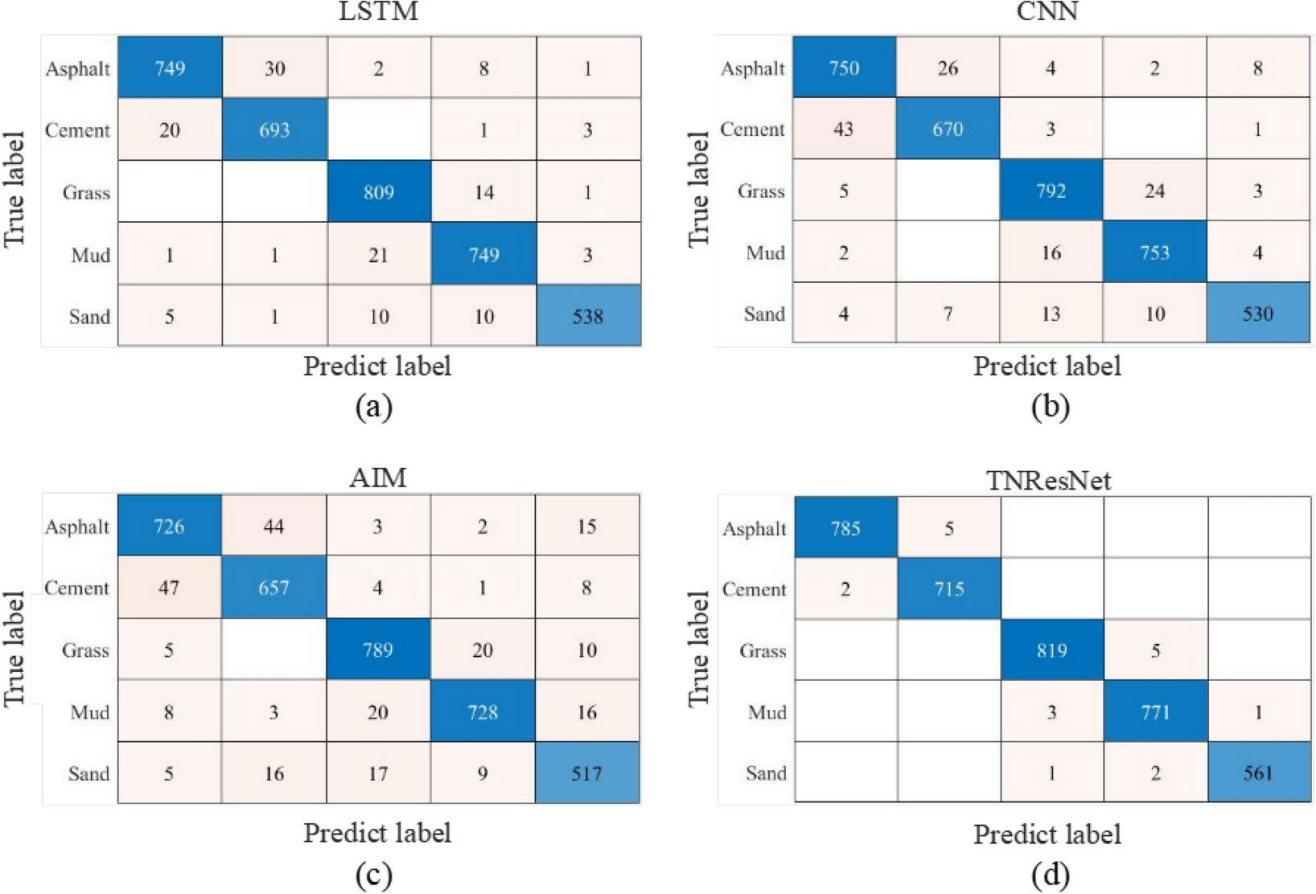
Confusion matrix for each best model of deep learning methods and TNResNet: (**a**) LSTM; (**b**) CNN; (**c**) AIM; (**d**) TNResNet. Deep learning-based methods have better performance than machine learning-based methods.

**Fig. 15:**
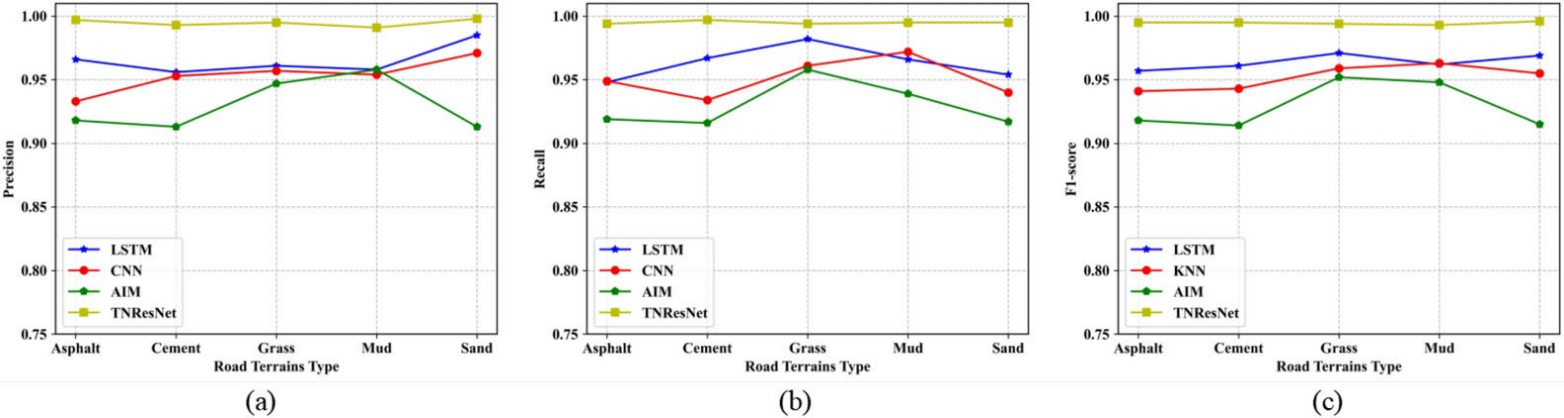
Evaluation metrics curve for each best model of deep learning methods and TNResNet: (**a**) precision; (**b**) recall; (**c**) F1-score. TNResNet has the best precision, recall and F1-score values and outperforms other compared deep learning methods.

The confusion matrices for each best model of deep learning methods and TNResNet are shown in Fig. 14. It’s easy to note that deep learning-based methods achieve significant boost performance over the machine learning-based methods. The precision, recall and F1-score values are displayed in Fig. 15 and listed in Supplementary Table S4, from which it can be found that the proposed TNResNet achieves the best precision, recall and F1-score values and outperforms other compared deep learning methods. Comparing to LSTM, CNN and AIM, there are 3.0%, 4.1%, 6.5% improvements in average precision; 3.2%, 4.4%, 6.5% improvements in average recall; and 3.1%, 4.3%, 6.6% improvements in average F1-score, respectively. While AIM shows the worst performance among these methods, which indicates that increasing the number of layers of the network can improve the road terrains recognition performance.

Enhancing precision indicates that TNResNet achieves greater precision in identifying road terrains across all categories. This enhanced accuracy suggests that the model has improved its ability to discern and categorize different road conditions effectively, crucial for the diverse environments encountered during autonomous vehicle operations.

Increasing the recall rate reduces the instances of missed critical terrain information, which is essential for autonomous vehicles during driving decision-making and route planning. For instance, ensuring that the vehicle control systems are correctly adjusted for different terrains can significantly mitigate the risk of potential accidents. This highlights the importance of a model that reliably identifies varying road conditions to ensure safety and efficiency.

A high F1-score indicates that TNResNet maintains a balance between high recall and high accuracy. This balance ensures that the system neither misses critical terrain features nor overidentifies non-target terrains. Such equilibrium is crucial for optimizing the performance of autonomous driving systems, ensuring that they operate effectively without unnecessary adjustments or errors in terrain recognition, thus enhancing both safety and operational efficiency in real-world conditions.

### Experimental results analysis

The comparison of TNResNet with traditional machine learning methods (such as Decision Trees, KNN, and SVM) and other deep learning models (including LSTM and CNN) highlights its significant advantages and innovations in diverse conditions.

In the evaluation against machine learning approaches, TNResNet demonstrates a remarkable improvement in road surface recognition tasks. The Decision Tree (DT) model achieves a maximum validation accuracy of 82.7%, while KNN and SVM attain maximum accuracies of 78.7% and 86.8%, respectively. However, TNResNet surpasses all these methods, illustrating its effectiveness in complex pattern recognition. Specifically, TNResNet outperforms DT, KNN, and SVM by enhancing average precision, recall, and F1 scores by 16.2%, 23.2%, 12.5%; 16.8%, 23.5%, 12.7%; and 16.6%, 23.4%, and 2.6%, respectively. These results indicate that TNResNet’s capability to extract high-level features enables it to better adapt to diverse road conditions.

When compared to deep learning models, TNResNet continues to excel. It consistently achieves superior performance across various evaluation metrics compared to LSTM and CNN. Notably, TNResNet shows improvements of 3.0%, 3.2%, and 3.1% in average precision, recall, and F1 score, respectively, over LSTM. This enhancement underscores TNResNet’s unique architecture in feature extraction and pattern recognition, making it more reliable for complex applications such as autonomous driving.

Furthermore, TNResNet’s design not only exhibits superior accuracy but also demonstrates exceptional adaptability. Its multi-layer network architecture effectively processes tire noise signals in varying environments, as validated by the experimental results. Whether on sandy, muddy, or concrete surfaces, TNResNet consistently displays high stability and accuracy. This adaptability meets the requirements of autonomous driving systems in dynamic environments, thereby improving driving safety and decision-making efficiency.

The high F1 score of TNResNet signifies a well-maintained balance between recall and precision. This balance is crucial for ensuring the system’s efficiency and accuracy in identifying critical road surface features, thus mitigating the risks of potential accidents and optimizing the overall performance of autonomous driving systems.

In summary, TNResNet not only proves its superiority in road surface recognition tasks but also provides a solid foundation for future research and applications in related fields through its innovative design and robust adaptability.

## Discussion

In this section, we address key considerations regarding the TNResNet model’s applicability, computational efficiency, and generalization capabilities for road terrain recognition in autonomous vehicles.

One of the critical aspects of TNResNet’s long-term viability is its operational stability and adaptability under varied environmental conditions. Our longitudinal simulations reveal fluctuations in model performance during extreme weather events, underscoring the need for enhanced training with weather-specific datasets. Implementing adaptive retraining mechanisms will be essential for maintaining sustained accuracy. Additionally, while TNResNet demonstrates high classification accuracy on common terrains, an analysis of misclassified data has revealed specific scenarios-such as unusual road textures and transitions-that lead to errors. Identifying these causes will inform future improvements at both the data pre-processing and model optimization levels.

Regarding computational efficiency, the demands of TNResNet on edge devices, typical in autonomous vehicles, necessitate further discussion. Strategies such as model pruning, quantization, and knowledge distillation can be employed to optimize the network for real-time processing, effectively reducing computational load while maintaining performance.

Furthermore, the experimental validation of TNResNet currently focuses on five common road surfaces, which limits its robustness demonstration. To enhance the model’s applicability, we recommend conducting additional tests on more challenging terrains, including snow, ice, and gravel. This diversity in terrain types will provide a more comprehensive understanding of the model’s generalization capabilities.

While our findings highlight the strengths of TNResNet, it is essential to acknowledge its limitations. The model’s performance on unmarked and degraded road surfaces has been inconsistent, suggesting a potential lack of generalization in unfamiliar conditions. Expanding the training datasets to encompass a wider range of terrain types and employing transfer learning techniques could enhance TNResNet’s robustness and adaptability.

To further refine TNResNet’s utility in autonomous driving applications, we recommend the following research directions:

1. Analysis of Misclassifications: Conduct a thorough analysis to understand the causes of misclassified data, leading to targeted improvements in data pre-processing and model optimization.
2. Environmental Adaptability Testing: Regularly implement environmental simulation tests to prepare the model for diverse operating conditions, ensuring better performance in real-world scenarios.
3. Resource Optimization Research: Explore efficient computation techniques specifically designed for autonomous driving systems to align the model’s requirements with computational constraints.
4. Enhancement of Generalization Skills: Expand the training datasets to include a broader variety of road conditions and investigate machine learning techniques that support better transferability and learning from sparse data environments.

By addressing these considerations, we aim to enhance the TNResNet model’s effectiveness and reliability in real-world autonomous driving applications.

## Conclusion

In this study, we introduce TNResNet, an end-to-end tire noise recognition residual network for road terrain recognition using tire noise signals captured by a low-cost microphone. TNResNet’s innovative features include a residual architecture that enhances deep learning capabilities and attention mechanisms that focus on critical signal features. This design significantly improves accuracy in distinguishing subtle variations between different road surfaces.

TNResNet achieved an impressive validation accuracy of 99.48%, outperforming classical machine learning methods (Decision Trees: 82.7%, KNN: 78.7%, SVM: 86.8%) and other deep learning models (LSTM: 96.4%, CNN: 95.2%). Additionally, it exhibited superior precision, recall, and F1-scores, underscoring its effectiveness in reducing false positives and negatives.

The model’s adaptability is a key advantage, enabling it to perform well under varied environmental conditions and handle background noise effectively. Its diverse training dataset equips TNResNet to recognize terrains that were not explicitly included in training, enhancing its robustness.

By advancing the field of road terrain recognition through acoustic analysis, TNResNet fills gaps in existing literature that often rely on visual or sensor-based methods. This innovation not only offers insights into tire-road interactions but also demonstrates the potential for integrating multi-sensory data in autonomous vehicle systems.

Overall, the accurate recognition capabilities of TNResNet contribute to improved vehicle control systems, enhancing the safety and efficiency of autonomous driving. Its unique architecture and adaptability make it a valuable asset for future research and practical applications in the field.

## Supplementary Information

Supplementary Information.

## Data Availability

The datasets used and analysed during the current study available from the corresponding author on reasonable request.
