# Supplementary material for: Road terrain recognition based on tire noise for autonomous vehicle
Source: Sci Rep. 2024 Dec 28;14:30913. doi: 10.1038/s41598-024-81666-7 (PMC11680919; doi:10.1038/s41598-024-81666-7)
Supplement: Supplementary file 1 — Supplementary Information. [file 41598_2024_81666_MOESM1_ESM.pdf]

Supplementary Table

Table S1. Time length (unit: second) of tire noise signal for each road terrain at varying speed.

Speed (km/h)	Asphalt	Cement	Grass	Mud	Sand
10-20	1050	740	1870	1945	1630
20-30	1045	1345	750	930	1390
30-40	795	500	500	1000	995
40-50	500	500	500	/	/
50-60	560	500	500	/	/
60-70	1000	750	500	/	/
70-80	440	725	500	/	/
Total	5390	5060	5120	3875	4015

Table S2. Statistical Feature of tire noise signals.

Road Type	Asphalt	Cement	Grass	Mud	Sand
Variance	0.0118	0.0022	0.0036	0.0402	0.0684
Skewness	0.0758	0.0705	0.0316	0.0325	0.0037
Kurtosis	3.2375	3.3007	3.3139	3.3368	2.6248
Standard	0.1086	0.0472	0.0598	0.2005	0.2615
Max	0.3461	0.1675	0.2072	0.6368	0.7313
Min	-0.3135	-0.2001	-0.1939	-0.6061	-0.7541
Range	0.6596	0.3686	0.4011	1.243	1.4854
Mode	-0.0555	-0.0061	-0.0173	0.0071	0.0649
Median	-0.0005	-0.0015	0.0006	-0.0034	-0.0021
Mean-Square	0.0188	0.0022	0.0036	0.0402	0.0684
Shannon Entropy	6.8	9.029	7.57	8.4347	9.8615
Main Frequency value	93	98	103	131	223

Table S3. Evaluation metrics for each best model of machine learning methods and TNResNet.

Model	Classes	Precision	Recall	F1-score
DT	Asphalt	75.1%	80.4%	77.7%
	Cement	78.9%	74.5%	76.6%
	Grass	87.5%	86.3%	86.9%
	Mud	84.0%	87.7%	85.8%
	Sand	90.7%	84.4%	87.4%
	Average	83.2%	82.7%	82.9%
KNN	Asphalt	72.6%	70.1%	71.3%
	Cement	73.0%	76.6%	74.8%
	Grass	77.6%	80.6%	79.1%
	Mud	76.5%	76.6%	76.5%
	Sand	81.8%	75.9%	78.7%
	Average	76.3%	76.0%	76.1%
SVM	Asphalt	84.2%	85.8%	85.0%
	Cement	85.6%	83.7%	84.6%
	Grass	88.8%	89.0%	88.9%
	Mud	87.2%	88.4%	87.8%
	Sand	89.0%	87.2%	88.1%
	Average	87.0%	86.8%	86.9%
TNResNet	Asphalt	99.7%	99.4%	99.5%
	Cement	99.3%	99.7%	99.5%
	Grass	99.5%	99.4%	99.4%
	Mud	99.1%	99.5%	99.3%
	Sand	99.8%	99.5%	99.6%
	Average	99.5%	99.5%	99.5%

Table S4. Evaluation metrics for each best model of machine learning methods, deep learning methods and TNResNet.

Model	Classes	Precision	Recall	F1-score
LSTM 6	Asphalt	96.6%	94.8%	95.7%
	Cement	95.6%	96.7%	96.1%
	Grass	96.1%	98.2%	97.1%
	Mud	95.8%	96.6%	96.2%
	Sand	98.5%	95.4%	96.9%
	Average	96.5%	96.3%	96.4%
CNN 3	Asphalt	93.3%	94.9%	94.1%
	Cement	95.3%	93.4%	94.3%
	Grass	95.7%	96.1%	95.9%
	Mud	95.4%	97.2%	96.3%
	Sand	97.1%	94.0%	95.5%
	Average	95.4%	95.1%	95.2%
AIM	Asphalt	91.8%	91.9%	91.8%
	Cement	91.3%	91.6%	91.4%
	Grass	94.7%	95.8%	95.2%
	Mud	95.8%	93.9%	94.8%
	Sand	91.3%	91.7%	91.5%
	Average	93.0%	93.0%	92.9%
TNResNet	Asphalt	99.7%	99.4%	99.5%
	Cement	99.3%	99.7%	99.5%
	Grass	99.5%	99.4%	99.4%
	Mud	99.1%	99.5%	99.3%
	Sand	99.8%	99.5%	99.6%
	Average	99.5%	99.5%	99.5%
